# Acceptability of physiotherapists as primary care practitioners and advanced practice physiotherapists for care of patients with musculoskeletal disorders: a survey of a university community within the province of Quebec

**DOI:** 10.1186/s12891-016-1256-8

**Published:** 2016-09-21

**Authors:** Ariel Desjardins-Charbonneau, Jean-Sébastien Roy, Julie Thibault, Vincent T. Ciccone, François Desmeules

**Affiliations:** 1 Unité de recherche clinique en orthopédie/ Orthopaedic clinical research unit, Centre de recherche de l’Hôpital Maisonneuve-Rosemont (CRHMR), University of Montreal Affiliated Research, Montreal, Quebec Canada; 2 Department of Rehabilitation, Faculty of Medicine, Laval University, Quebec City, Quebec Canada; 3 Center for Interdisciplinary Research in Rehabilitation and Social Integration, Quebec City, Quebec Canada; 4 School of Rehabilitation, Faculty of Medicine, University of Montreal, Montreal, Quebec Canada

**Keywords:** Advanced practice, Physiotherapy, Musculoskeletal disorders

## Abstract

**Background:**

Musculoskeletal (MSK) disorders represent a great burden on the health care system. The use of physiotherapists in their autonomous roles and in advanced practice roles may help increase access to care. Thus, the aim of this survey was to assess the perceptions of a university community sample within the province of Quebec about physiotherapists as primary care practitioners and advanced practice physiotherapists (APPs) for the treatment of patients with musculoskeletal disorders.

**Methods:**

An electronic survey was sent in February 2014 via a web platform to members of the Laval University community (Québec City, Canada). The survey included questions about knowledge and perceptions on current physiotherapists’ autonomous role in primary care and on APP future model of care for patients with MSK disorders. Survey results were synthetized with descriptive statistics. Differences in responses according to demographics, personal characteristics and previous physiotherapy care experience were evaluated using Chi-Square tests.

**Results:**

A total of 513 participants completed the online survey (1 % response rate). The majority of respondents were women (74 %) and aged 18 to 24 (39 % of all respondent). About 90 % of respondents believed that physiotherapists were skilled and competent and 91 % answered that they had trust in physiotherapists for the treatment of MSK disorders in primary care. A total of 90 % of respondents supported the idea of introducing APPs for the treatment of patients with MSK disorders. Over 90 % of respondents were in favour of the delegation of medical acts such as: communicating a medical diagnosis, ordering imaging tests, triaging surgical candidates or prescribing medication such as NSAIDS.

**Conclusions:**

Respondents are satisfied and have confidence in physiotherapists as primary care practitioners; they also support the intended new roles of the APPs in the health care system. Caution should be taken in generalizing these results from this particular sample. These results need to be corroborated in the general population.

**Electronic supplementary material:**

The online version of this article (doi:10.1186/s12891-016-1256-8) contains supplementary material, which is available to authorized users.

## Background

Musculoskeletal (MSK) disorders, such as osteoarthritis, rheumatoid arthritis, osteoporosis and low back pain, represent an important burden in terms of direct and indirect health care costs in western countries [1, 2]. In Canada, pressure on the health care system is rising; the prevalence of MSK disorders is increasing, while access to care declines [3–6]. New models of care are needed to improve access and ameliorating care for this population. Mounting evidence supports the use of non-physician health professionals in more autonomous roles to help increase access in primary or secondary care [7]. In these new roles, non-physician health professionals are positioned upstream in the health care system and become primary care providers. Primary care providers typically provide first contact to persons with any undiagnosed disorder [8]. Physiotherapists are among the health professionals who are expected to play increasingly important roles as primary care providers for the treatment of MSK disorders as they have the competence and skills to diagnose and manage a variety of MSK disorders without any medical involvement [9–11]. In Canada, individuals that have a MSK disorders have access to physiotherapy care without a medical referral either in private practice or in publicly funded institutions. In Canada and elsewhere throughout the world, direct access to physiotherapy care has been associated with improved access, equal or better patient outcomes and decreased health care costs [7].

Apart from direct access primary care providers, physiotherapists may also work in an extended or advanced scope of practice more commonly called advanced practice physiotherapy (APP) in Canada. APP is gradually being implemented throughout Canada and elsewhere in the world [11, 12]. In Canada, APP includes role enhancements, role substitution related to traditionally performed medical acts or delegation of controlled acts [11]. In Canada, these models have typically been implemented in orthopaedic settings mostly in secondary care. These innovative models for physiotherapists have already shown important benefits in terms of access to care, efficacy and efficiency, but for a successful and sustainable implementation, patient satisfaction and acceptability of these new models is mandatory [11]. Indeed, there is an increasing need to offer patient-centred care where patients are given greater autonomy and responsibility for the choices of care they receive and from which provider it is offered; the patient perspective on these new models of care is thus required [13, 14].

In recent years in Canada, three surveys have asked Canadians about their knowledge and perceptions of physiotherapists [15–17]. The participants surveyed believed that physiotherapists were competent, that they delivered effective treatment and were also good communicators [15, 17]. One survey reported that the general perception on physiotherapy was more favourable in specific subgroups of the population according to factor such as: age (35–44), lifestyle (active population) or education level (university degree); [17] two surveys reported that although Canadians trust the competence of physiotherapists, they often prefer to see a family physician first when suffering from a MSK disorder [16, 17]. While these surveys detailed certain perceptions of the public regarding physiotherapy, they did not assess specifically the population’s perceptions on the autonomous role of physiotherapists as primary care practitioners and on the APP model of care. Moreover, only one of those surveys was done in the province of Québec and was done, almost 5 years ago in 2011. Since the perception of the physiotherapy professional practice by the population is important as it may facilitate or impede changes to occur in the profession, it is important to have an up-to-date perception of the public on these matters. Thus, our objective was to assess the perceptions of a university community sample within the province of Quebec about physiotherapists as primary care practitioners and advanced practice physiotherapists (APPs) for the treatment of patients with MSK disorders.

## Methods

This descriptive study used a cross-sectional design and was approved by the Laval University Research Ethic Board (Le Comité d’éthique de la recherche avec des êtres humains de l’Université Laval, 2014-007/07-02-2014), in Québec City, Canada.

### Target population

This survey used a convenience sample; an electronic invitation was sent to all members of the Laval University community in Quebec City, Canada via email. The Laval University community has 52,100 registered members with a valid email address. Therefore students, professors, teaching assistants, researchers, support staff, administration and direction members constituted the survey sample and were eligible participants. Being a member (teacher or support staff) or a student of the Department of Rehabilitation was the only exclusion criterion. Individuals participated voluntarily. Because of institutional regulation, no reminders were sent to complete the survey. No compensation was offered to participants.

### Survey questionnaire and data collection

The survey was developed in French following a review of the literature on APP [11] and was based on content of previous surveys made by professional Canadian physiotherapy associations/colleges regarding acceptability of the profession or of anticipated new roles for physiotherapists such as APP [15–21]. The survey included questions regarding: 1- diagnostic ability of physiotherapist 2- efficacy and safety of care, 3-satisfaction with care, 4- effects on access to care and use of health care resources. More precisely, the survey, hosted on the *Surveymonkey.com* platform, had 37 questions divided in four sections: 1- Previous physiotherapy care and satisfaction with previous episode of care either by a physiotherapist or physical rehabilitation technician (Q2-10), 2- knowledge and perceptions on current physiotherapists’ autonomous role in primary care management of patients that have a MSK disorders (Q11-19), 3- perceptions on APP future model of care for patients with MSK disorders (Q20-31) and 4- participants’ demographic characteristics (Q32-37) (Additional files 1 and 2). MSK disorders were defined with examples such as low back pain, neck pain, sprain, strain, tendinitis, muscular and joint pain [22]. Information on APP model of care was provided at the beginning of the survey to inform participants of this new model of care. This information included the description of the new roles, of the additional training, the targeted conditions and the advantages it might provide based on evidence from other countries and other Canadian provinces (Additional files 1 and 2). The questionnaire used multiple choice questions and 4 or 5-point Likert scale response options. The survey was pilot-tested by five selected respondents (aged between18-65, two of them had a university degree and some medical knowledge) to evaluate the clarity and precision of the questions and answers; clarifications of questions were made based on their suggestions. An email that included the link to complete the survey (active for 2 weeks) and information on the time required to complete the survey (about 30 min) was sent in February 2014. No personal data that could identify participants were collected in the survey. The Surveymonkey.com web platform is secure, using SSL/TLS encryption to protect data.

### Data analysis

Raw data was exported into an Excel spreadsheet (Microsoft Corp., Redmond, WA) and, missing data was assessed. Participants who did not complete 80 % of the questionnaire were excluded from the analyses. Descriptive analysis was performed on the remaining sample. When questions were on a 5-point Likert scale, data was merged from the first 2 and last 2 categories, thus leaving the scale on a 3-point form to facilitate categorical statistical testing. Chi square tests (χ^2^) were performed to compare responses according to demographic characteristics (age, sex or occupation) and previous history of physiotherapy care. Z-tests were performed to compare proportions for age and occupation because it included more than two categories. The alpha level was set at 0.05. For Z-tests on age and occupation, a Bonferroni corrections was applied because it included more that 2 comparaisons. Statistical analyses were performed with SPSS (v.21, SPSS Inc., Chicago, IL).

## Results

Of the 52,000 potential participants, 589 participants agreed to participate and respond to the survey. The overall response rate was 1 % and the completion rate was 87 %. Data from 76 participants were excluded for the following reasons: 1- Staff or student of the Rehabilitation Department (*n* = 13), 2- less than 80 % of the questionnaire was completed (*n* = 43) and 3- demographic characteristics were missing (*n* = 20). Analyses were therefore performed on the remaining 513 participants. Some questions focused on care received by physical rehabilitation technicians or types of other health conditions that a physiotherapist treats, results from these questions are not reported here as they are not the main objective of this paper.

Respondents were predominantly women (74 %) and more than half (55 %) fell into the 18-29 age category. The sample mostly consisted of students (64 %) and university support staff (15 %). Around two thirds of the respondents (64 %) had previously consulted a physiotherapist (Table 1).

**Table 1:** Participant characteristics according to previous physiotherapy treatment (*n* = 513)

	Participants with previous physiotherapy care (*n* = 329)	Participants without previous physiotherapy care (*n* = 184)
Sex		
Male	89 (27 %)	44 (24 %)
Female	240 (73 %)	139 (76 %)
Age		
18–29	154 (47 %)	129 (71 %)
30–39	63 (19 %)	33 (18 %)
40–49	48 (15 %)	12 (7 %)
50+	64 (19 %)	10 (5 %)
Occupation^a^		
Student	186 (57 %)	142 (77 %)
Support or technical personnel	57 (17 %)	18 (10 %)
Managing position	42 (13 %)	13 (7 %)
Researcher/ professor	37 (11 %)	9 (5 %)
Others	4 (1 %)	2 (1 %)
Marital status^b^		
Married or common law	108 (33 %)	44 (24 %)
Single	202 (61 %)	132 (72 %)
Divorced, separated or widower	10 (3 %)	7 (4 %)
Educational level completed		
College^c^	131 (40 %)	87 (47 %)
University, undergraduate	90 (27 %)	50 (27 %)
University, postgraduate	97 (29 %)	42 (22 %)
Primary or secondary school	11 (3 %)	0 (0 %)
Native language		
French	319 (97 %)	178 (97 %)
English	4 (1 %)	2 (1 %)
Others	6 (2 %)	4 (2 %)

^a^ *n* = 506

^b^ *n* = 507

^c^College degree in the province of Quebec includes the 12^th^ year of high school and the first year of an associate university degree

### Knowledge and perceptions on Physiotherapists’ autonomous role in primary care management of patients with MSK disorders

**Fig. 1:**
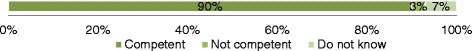
What is your opinion about the skills and competence of physiotherapists? (*n* = 513)

**Fig. 2:**
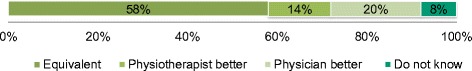
If you are suffering from back or neck problems, from a sprain, a tendinitis or you have muscle or joint pain, do you believe that a physiotherapist can make a diagnosis equivalent to the one of a family or an emergency doctor? (*n* = 513)

Sixty-four percent (*n* = 329) of respondents had previously consulted a physiotherapist and a high proportion (84 %) of them were satisfied (satisfied or very satisfied) with treatments they had received. Among all respondents, 90 % (*n* = 461) reported that they felt physiotherapists were competent and skilled (competent and skilled or very competent and skilled) (Fig. 1) and 93 % (*n* = 464) said they trusted (confident or very confident) the quality of the treatment provided by physiotherapists for patients with MSK disorders. Nearly three quarters (*n* = 369) of respondents reported that the ability of a physiotherapist in its usual role for the diagnosis of MSK disorders is equivalent to if not better than the ability of a family physician or an emergency room physician (Fig. 2). When asked for the necessity of a physicians’ diagnosis before the initiation of physiotherapy care, 58 % (*n* = 297) believed that it was not mandatory (little essential or not essential at all). This proportion was significantly higher in the group of respondents that had undergone previous physiotherapy treatments (63 % compared to 50 %, *p* = 0.007). However, nearly 12 % (*n* = 60) of respondents believed that it is mandatory to obtain a medical referral before seeing a physiotherapist, while another 11 % (*n* = 56) were not aware that physiotherapists are allowed direct access. Again, when taking into account previous treatment history, the proportion of respondents who did not think that a medical reference was mandatory was different in both groups (84 % for the group of respondents that had undergone previous physiotherapy treatments compared to 65 % for those who had not, *p* < 0.001).

**Fig. 3:**
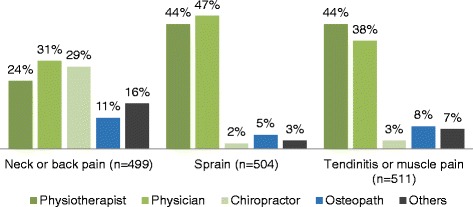
Which health care provider would you consult first?

When asked which health care professional the participants would consult first for specific MSK conditions, results varied by conditions and previous experience with physiotherapy. Physiotherapists were the health professionals most likely to be consulted for the treatment of tendinitis or muscle pain (44 %), compared to family physicians (38 %), chiropractors (3 %) or osteopaths (8 %). However, for a joint sprain, family physicians (47 %) were the preferred professionals to first consult, followed by physiotherapists (44 %), osteopaths (5 %) and chiropractors (2 %). For back or neck pain, 31 % of participants would consult a family physician first, 29 % a chiropractor, 24 % a physiotherapist and, 11 % an osteopath (Fig. 3). For all these MSK conditions, participants who had received previous physiotherapy care were more likely to consult a physiotherapist first (*p* < 0.05). These results did not significantly differ according to sex, (*p* ≥ 0.05) age (*p* ≥ 0.008; Bonferoni corrected alpha level) or occupation(*p* ≥ 0.008; Bonferoni corrected alpha level). A total of 31 % (*n* = 153) of respondents believed that imaging tests (X-rays, MRI or other tests) are necessary to confirm an MSK diagnosis and 17 % (*n* = 86) responded that they are not always necessary but still prefer to have them prescribed. In terms of treatment, 92 % (*n* = 459) believed that it is not always necessary to take prescription drugs such as analgesics and non-steroidal anti-inflammatory drugs (NSAID) to effectively treat an MSK disorder.

### Perceptions on APP model of care

**Fig. 4:**
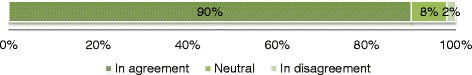
APP is an overall favourable phenomenon? (*n* = 513)

**Fig. 5:**
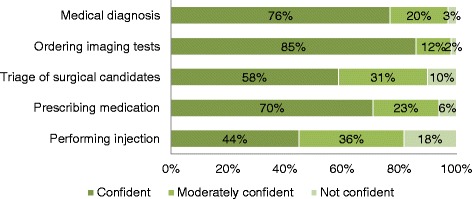
Confidence level in medical act delegation to an advanced practice physiotherapist (*n* = 513)

The vast majority of respondents believed that APP is a favourable phenomenon (favourable or very favourable) (90 %, *n* = 461) (Fig. 4) and that treatments by APPs would be safe (safe or very safe) (96 %, *n* = 492). Five questions assessed the confidence level in the delegation of medical acts to APPs for care of patients with MSK disorders. Most respondents stated that they would trust (very or extremely confident) the competence and skills of APPs to make a valid medical diagnosis (76 %, *n* = 390), ordering medical imaging tests (85 %, *n* = 436), triaging patients for surgical care (58 %, *n* = 298) and prescribing medication such as NSAID (70 %, *n* = 359). The only medical act in which participants had less confidence was injections: 44 % (*n* = 226) of respondents had confidence, 36 % (*n* = 185) had a moderate level of confidence and 18 % (*n* = 92) had only a little confidence in APPs performing injections (Fig. 5). Respondents were also comfident (very or extremely confident) with APPs referring patients to a family physician when required (83 %, *n* = 426) and more generally that APPs would make adequate decisions regarding their health (63 %, *n* = 323). Only 20 % (*n* = 103) of respondents believed (agree or strongly agree) that family physicians have essential knowledge that APPs would not have. In terms of access to care, the majority of respondents believed that the implementation of APPs would reduce wait times before being assessed or treated (72 %, *n* = 369) and would reduce the hospital length of stay for admitted patients (56 %, *n* = 287). Responses to all questions from this section did not differ according to sex (*p* ≥ 0.05), age (*p* ≥ 0.008; Bonferoni corrected alpha level), occupation (*p* ≥ 0.008; Bonferoni corrected alpha level) or previous physiotherapy care (*p* ≥ 0.05).

## Discussion

The purpose of this study was to assess the perception of a sample of the population regarding the role of physiotherapists in primary care management of MSK disorders and the acceptability of the APP model of care. Perceptions of the physiotherapy profession and new roles such as the APP are mandatory for evidence-based medicine. Positive perceptions could impact future health care delivery by favouring a quicker and broader implementation of APP. The overall perception from a sample from a single academic community within Quebec’s population is that physiotherapists are competent and that the acceptability of APPs is high. These results should be interpreted cautiously as the survey results are based on an electronic survey from a convenience sample in one university in the province of Quebec, Canada. Nonetheless, we believe these results provide valuable information regarding the acceptability of emerging physiotherapy roles in primary care for MSK disorders.

Concerning the public’s knowledge and perceptions of physiotherapists’ autonomous role for primary care management of MSK disorders, our results show that respondents perceived physiotherapists as competent, whether or not they had already consulted with a physiotherapist in the past. This is in line with results from an online survey made in Ontario, another Canadian province, in 2011 for the Ontario Physiotherapy Association (*n* = 1,004) which aimed at assessing the adult population’s knowledge and satisfaction regarding the physiotherapy profession (this survey was weighted by region, age and gender according to the Census data to improve its external validity) [16]. As in our survey, the respondents perceived that the physiotherapists’ diagnoses for MSK disorders were as valid, if not more valid, than the diagnoses made by medical providers. Still, in our survey, it appears that the current role of physiotherapists is not always well understood, and the choice to consult a family physician first remains predominant for an important proportion of respondents. This finding may have to do with the fact that not everyone has access to private health insurance to cover costs associated with physiotherapy services, or that some insurance companies require a medical prescription to reimburse physiotherapy treatments. It may also be associated with the fact that a proportion of respondents did not know that physiotherapists have direct access to care. However, this behaviour was significantly higher in the subgroup of patients who had never received physiotherapy care, suggesting that once patients had experienced physiotherapy care they are more likely to use direct access the next time. Again, this result is similar to the 2011 Ontarian survey, where 41 % of the respondents reported consulting their family physician first before consulting in physiotherapy [16]. These results also suggest that the physiotherapy profession should potentially increase the promotion of their capacity as primary practitioners and as APPs to the public and to health care policy makers.

Still, our results and the results of the Canadian survey done in the province of Ontario are different from a third Canadian online survey ordered by the Physiotherapy Association of the Canadian province of British Columbia (adults, *n* = 824), in which 70 % of respondents believed that a referral from a physician was mandatory [15]. The disparity may come from the fact that their sample was different: participants were selected from a public opinion database where they were previously enrolled and received compensation for the completion of the survey. Their sample was also statistically weighted according to Canadian census figures.

It is important to assess the perception about the necessity of imaging tests and medication prescription in the treatment of MSK disorders because, in their usual role, physiotherapists may not be permitted to perform these acts depending on regulatory bylaws where they practice. If patients have the perception that these acts are necessary for optimal care, they might prefer to see a physician before engaging in physiotherapy care. In our sample, results suggest that respondent did not have major misconceptions in terms of the appropriateness of imaging tests needed to confirm a MSK disorder or prescription of medication, only 31 % believe that imaging tests are necessary to confirm an MSK diagnosis and only 8 % believe that prescription drugs are necessary to treat a MSK disorder. However, these results should be viewed cautiously as our sample was from a university setting where respondents are likely to be more health literate than the average population.

On the perceptions of the APP model, our results show that the vast majority of respondents thought the implementation of APPs would be a favourable and safe phenomenon. Our results are in line with other surveys assessing the acceptability of the delegation of medical roles to other health professionals such as physiotherapists [11] and nurses [23]. The majority of respondents had confidence in the delegation of medical acts to APPs for care of patients with MSK disorders. They were also comfortable with the idea of a physiotherapist referring a patient to a family physician only if needed and they trusted that they would make adequate decisions regarding their health. According to our results, respondents believed that the implementation of an APP model would reduce the waiting time and the duration of hospital lengths of stay for admitted patients. Indeed, in the Canadian healthcare system, some APPs can provide post-surgical follow- up for patients with MSK disorders, in this context the discharge for hospitalized patients could be quicker with APP care resulting in a shorter length of stay. It is interesting to acknowledge that no significant differences were observed between respondents that had had previous physiotherapy care and those who had not. According to our survey, physiotherapists are considered competent and this may explain why people are open to the implementation of APP models even if they never had previous physiotherapy care. Finally, our results provide growing evidence to governments and health agencies that the public are supportive of new initiatives that can reduce the burden on the medical profession and increase access to safe efficient care. Still, in this survey we did not specifically address the required training and the competence needed by APPs to provide efficient and safe care in the province of Québec, future implementation of APP will be made with adequate physiotherapists’ training updates. Further studies should also investigate the acceptability of physiotherapists as primary care providers and of the APP model of care in other settings to have a broader insight of the population’s perception so health care could be tailored to the population’s needs and be better delivered.

## Strengths and limitations

The strengths of this survey were its relatively large sample size of participants and the fact that it was the first to document perception and acceptability of physiotherapists in their usual primary care role and of APP models of care in Canada. However, the participation rate was small and the use of an anonymous online survey using closed questions have limitations and other survey methodology could have yielded different results. In addition, our sample was composed of a Quebec university’s community members aged mostly between 18 and 29 and highly educated, therefore this sample may not reflect the general population, and so we must be cautious about generalizing these results to Quebecer or Canadians. Nonetheless, this survey provides encouraging positive results that support the implementation of APP models.

## Conclusion

Our results suggest that respondents are quite satisfied with and have confidence in physiotherapists in their usual roles for the primary care management of MSK disorders. Respondents are also receptive to the introduction of the APP for patients with MSK disorders. Our results can not be generalized to all Canadians since the survey was conducted among people in an academic community, but we do believe that it constitutes very encouraging data, which tends to support the implementation of APP. The implementation of the APP model of care should continue across the country to further evaluate the efficiency, safety and acceptability of this new model.

## Key messages

### What is already known on this topic

Physiotherapists in their usual roles are primary care providers for the treatment of MSK disorders. Advanced practice physiotherapy (APP) is a new model of care, which is gradually being implemented throughout the world. Advanced practice physiotherapists (APPs) may work as primary care or secondary care practitioners with medical delegated acts to further extend their autonomy.

### What this study adds

In the university-based sample surveyed, respondents are satisfied with and have confidence in physiotherapists as primary care practitioners. They are also receptive to the introduction of APPs in the health care system.

## Additional files

Additional file 1: Original (french) version of the survey. (DOCX 109 kb) Additional file 2: English translation of the survey. (DOCX 111 kb)

## Abbreviations

APP: Advanced practice physiotherapy
APPs: Advanced practice physiotherapists
MSK: Musculoskeletal
NSAID: Non-steroidal anti-inflammatory drugs
