# Supplementary material for: Acceptability of physiotherapists as primary care practitioners and advanced practice physiotherapists for care of patients with musculoskeletal disorders: a survey of a university community within the province of Quebec
Source: BMC Musculoskelet Disord. 2016 Sep 21;17:400. doi: 10.1186/s12891-016-1256-8 (PMC5031271; doi:10.1186/s12891-016-1256-8)
Supplement: Additional file 1: — Original (french) version of the survey. (DOCX 109 kb) [file 12891_2016_1256_MOESM1_ESM.docx]

**Appendix 1**

Mise en contexte

Cette étude est une étude quantitative de type sondage portant sur la perception du public quant à la physiothérapie. Toutes vos réponses sont confidentielles et aucune information permettant de vous identifier ne sera recueillie. Le sondage prend environ 15 minutes à remplir. Le questionnaire sera divisé en deux parties. La première évaluera votre niveau de perception et votre niveau de connaissance par rapport à la physiothérapie. L’émergence de la profession de physiothérapeute dans les dernières décennies en fait maintenant une profession importante et il est important de sonder la population quant à leur perception de cette profession. La deuxième partie du sondage évaluera votre perception par rapport à la pratique avancée en physiothérapie au Québec. On voit l’émergence de nouveaux rôles pour les physiothérapeutes ayant pour but d’améliorer l’accessibilité et l’efficience des soins de santé pour les clientèles souffrant de problèmes d’arthrose, de tendinites ou de maux de dos. Ce sondage est réalisé par un chercheur de l’Université Laval, Jean-Sébastien Roy en collaboration avec des étudiants à la maîtrise en physiothérapie ainsi qu’un chercheur de l’Université de Montréal, François Desmeules.

1. Travaillez-vous au département de réadaptation ou étudiez-vous au programme de physiothérapie de l’Université Laval ?

A. Oui 􏰀 Merci de votre réponse, mais vous ne pouvez participer à ce sondage

B. Non

***Partie 1 : Perception du public sur la physiothérapie comme intervenante de première ligne dans le traitement de problèmes musculosquelettiques divers.***

2. Avez-vous déjà eu recours aux services d’un physiothérapeute ou d’un thérapeute/technicien en réadaptation physique?

- Oui
- Non 􏰀Passage à 7
- Je ne sais pas 􏰀Passage à 7

3. Vous avez reçu ces traitements en physiothérapie (cocher toutes les cases nécessaires) :

1. Dans une clinique privée
2. Dans un hôpital
3. Via le CLSC
4. Dans un centre de réadaptation
5. Lors d’un événement sportif
6. Autre endroit : Préciser :___________

4. À quand remonte votre dernière visite en physiothérapie?

1. Au cours de la dernière année
2. Il y a plus de 1 an
3. Il y a de 2 à 5 ans
4. Il y a plus de 5 ans
5. Il y a plus de 10 ans

5. Quel a été votre niveau de satisfaction suite aux traitements reçus en physiothérapie?

Si vous avez reçu des traitements pour différents problèmes de santé à plus d’une reprise, veuillez considérer votre dernière série de traitements pour un même problème de santé.

1. Très satisfaisant
2. Plutôt satisfaisant
3. Peu satisfaisant
4. Très peu satisfaisant
5. Je ne sais pas

6. Il existe deux classes de professionnels qui peuvent donner des soins en physiothérapie : le physiothérapeute possédant une formation universitaire ou le thérapeute ou technicien en réadaptation physique qui possède une formation collégiale. Lequel de ces professionnels vous a donné des traitements lors de vos visites les plus récentes?

1. Un physiothérapeute
2. Un thérapeute ou technicien en réadaptation physique
3. Les deux
4. Je ne sais pas quel professionnel m’a donné des traitements

7. SI vous avez eu besoin dans le passé de consulter un physiothérapeute ou si dans le futur vous auriez à le faire, comment allez-vous ou l’avez-vous choisi ?

1. Suivant les conseils de votre médecin
2. Suivant les conseils d’un membre de votre famille ou un ami
3. Par rapport à des critères de proximité (près de chez vous ou de votre travail)
4. Selon des informations, des messages publicitaires sur internet, dans les  médias ou dans les *pages jaunes*
5. Autre raisons. Précisez:
6. Je ne sais pas

8. Quelle est votre opinion sur la compétence des physiothérapeutes ?

1. Très compétents
2. Compétents
3. Peu compétents
4. Très peu compétents
5. Je ne sais pas

9. Quelle est votre opinion sur la compétence des thérapeutes ou techniciens en réadaptation physique ?

1. Très compétents
2. Compétents
3. Peu compétents
4. Très peu compétents
5. Je ne sais pas

10. Avez-vous confiance en la qualité des traitements reçus ou que vous pourriez recevoir en physiothérapie?

1. Très confiant
2. Confiant
3. Peu confiant
4. Très peu confiant
5. Je ne sais pas

43

11. Selon vous quels sont les problèmes de santé qu’un physiothérapeute peut prendre en charge (évaluer et traiter)? (cochez tous les problèmes qui s’appliquent)

1. Musculosquelettiques (ex:entorse, tendinite, maux de dos et du cou)
2. Incontinence urinaire (chez la personne âgée ou chez la femme post-  grossesse)
3. Troubles de l’équilibre (chute fréquente secondaire à une condition  neurologique)
4. Affections neurologiques (accident vasculaires cérébral)
5. Trouble à la marche
6. Affections respiratoires (pneumonie)
7. Pédiatriques (problèmes de développement physique chez l’enfant)

12. Si vous souffrez de maux de dos ou de cou, d’une entorse, d’une tendinite ou de douleurs musculaires ou aux articulations, croyez-vous que le **physiothérapeute** peut poser un diagnostic équivalent à un médecin de famille ou un médecin à l’urgence ?

1. Oui, les physiothérapeutes et les médecins peuvent poser des diagnostics valides et précis équivalents
2. Non, je crois que le diagnostic du médecin est plus valide et précis
3. Non, je crois que le diagnostic du physiothérapeute est plus valide et précis
4. Je ne sais pas

13. Si vous souffrez de maux de dos ou de cou, d’une entorse, d’une tendinite, de douleurs musculaires ou aux articulations, croyez-vous qu’il est toujours nécessaire de faire une radiographie, une résonnance magnétique ou un autre test radiologique pour faire un diagnostic valide?

1. Oui
2. Non, ces tests ne sont pas toujours nécessaires
3. Non, mais je préfère avoir une confirmation du diagnostic avec un de ces  tests.
4. Je ne sais pas

14. Si vous souffrez de maux de dos ou de cou, d’une entorse, d’une tendinite, de douleurs musculaires ou aux articulations, Croyez-vous qu’il est toujours nécessaire de prendre des médicaments sous ordonnance pour traiter efficacement ces problèmes ?

1. Oui
2. Non, des médicaments ne sont pas toujours nécessaires
3. Non,mais ils accélèrent la guérison
4. Je ne sais pas

15.Avant que le physiothérapeute vous prenne en charge (évaluation et traitement), l’avis d’un médecin est :

1. Absolument essentiel
2. Essentiel
3. Peu essentiel
4. Pas du tout essentiel

16.Selon-vous, un patient doit-il avoir une référence médicale pour aller consulter un physiothérapeute dans une clinique privée?

1. Oui
2. Non
3. Je ne sais pas

17. Quel intervenant consultez-vous ou consulteriez-vous **en premier** pour des maux de dos ou de cou?

1. Médecin
2. Chiropraticien
3. Kinésiologue
4. Physiothérapeute
5. Massothérapeute
6. Ostéopathe
7. Autre : Préciser :________________  18. Quel intervenant consultez-vous ou consulteriez-vous **en premier** pour une entorse aux niveaux des bras ou des jambes?
8. Médecin
9. Chiropraticien
10. Kinésiologue
11. Physiothérapeute
12. Massothérapeute
13. Ostéopathe
14. Autre : Préciser :________________  19. Quel intervenant consultez-vous ou consulteriez-vous **en premier** pour une tendinite, des douleurs musculaires ou des douleurs aux articulations des bras ou des jambes?
15. Médecin
16. Chiropraticien
17. Kinésiologue
18. Physiothérapeute
19. Massothérapeute
20. Ostéopathe
21. Autre : Préciser :________________

**Partie 2 : Perception du public sur la pratique avancée en physiothérapie**

La deuxième partie du questionnaire porte sur la pratique avancée en physiothérapie. Il s’agit de nouveaux modèles de soins où des **physiothérapeutes ayant une formation supplémentaire** se voit accorder des actes normalement réservés aux médecins tels que le droit de poser un diagnostic médical, évaluer des patients pour déterminer si une chirurgie orthopédique est nécessaire, demander des tests d’imagerie (radiographie, IRM par exemple) et dans certains cas prescrire/injecter de la médication chez des patients souffrant de maux de dos ou de cou, d’une entorse, d’une tendinite ou de douleurs musculaires ou des douleurs aux articulations. En offrant plus d’autonomie aux physiothérapeutes en pratique avancée, on permet en autre de désengorger le système de santé. Ces physiothérapeutes en pratique avancée, suivant une formation complémentaire, pourraient être implantés dans le futur au Québec. C’est un phénomène semblable à celui des infirmières praticiennes, souvent appelées super- infirmières.

Les questions qui suivent, se rapportent à la pratique avancée en physiothérapie pour la prise en charge et le traitement de patients souffrant de maux de dos ou de cou, d’une entorse, d’une tendinite ou de douleurs musculaires ou de douleurs aux articulations, d’arthrose ou d’autres problèmes musculosquelettiques (c’est à dire des muscles, des os, des articulations et des tendons)

Les questions suivantes vous demandent d’indiquer votre niveau de confiance par rapport à différents aspects de la physiothérapie en pratique avancée, si elle était implantée ici au Québec.

20. Le physiothérapeute en pratique avancée déterminerait votre diagnostic et dans la grande majorité des cas, vous ne rencontreriez pas le médecin. Quel serait votre degré de confiance envers un physiothérapeute en pratique avancée dans ce rôle ?

1. Pas confiant du tout
2. Pas très confiant
3. Confiance modérée
4. Très confiant
5. Extrêmement confiant
6. Je ne sais pas

21.C’est le physiothérapeute en pratique avancée qui demanderait les tests radiologiques jugés nécessaires pour évaluer votre condition (radiographie, résonnance magnétique ou autres tests radiologiques). Quel serait votre degré de confiance envers un physiothérapeute en pratique avancée dans ce rôle ?

1. Pas confiant du tout
2. Pas très confiant
3. Confiance modérée
4. Très confiant
5. Extrêmement confiant
6. Je ne sais pas

22. Le physiothérapeute en pratique avancée déterminerait si vous avez besoin d’une chirurgie pour le traitement de votre problème de santé musculosquelettique (des muscles, des os, des articulations ou des tendons) et ensuite, il vous réfèrerait pour rencontrer le chirurgien et déterminer exactement la chirurgie à faire. Quel serait votre degré de confiance envers un physiothérapeute en pratique avancée dans ce rôle ?

1. Pas confiant du tout
2. Pas très confiant
3. Confiance modérée
4. Très confiant
5. Extrêmement confiant
6. Je ne sais pas

23.Le physiothérapeute en pratique avancée pourrait vous prescrire certains médicaments comme des anti-inflammatoires. Quel serait votre degré de confiance envers un physiothérapeute en pratique avancée dans ce rôle ?

1. Pas confiant du tout
2. Pas très confiant
3. Confiance modérée
4. Très confiant
5. Extrêmement confiant
6. Je ne sais pas
7. Le physiothérapeute en pratique avancée pourrait procéder à des injections dans les muscles ou les articulations. Quel serait votre degré de confiance envers un physiothérapeute en pratique avancée dans ce rôle ?
   1. Pas confiant du tout
   2. Pas très confiant
   3. Confiance modérée
   4. Très confiant
   5. Extrêmement confiant
   6. Je ne sais pas
8. Le fait d’être pris en charge par un physiothérapeute plutôt que par un médecin me permettrait d’attendre moins longtemps pour un rendez-vous ou pour un traitement.
   1. Tout à fait en accord
   2. En accord
   3. Ni accord ni en désaccord
   4. Pas d’accord
   5. Pas du tout d’accord
9. Le fait d’être pris en charge (évaluation et traitement) par un physiothérapeute plutôt que par un médecin à l’hôpital me permettrait d’y passer un séjour plus court.
   1. Tout à fait en accord
   2. En accord
   3. Ni accord ni en désaccord
   4. Pas d’accord
   5. Pas du tout d’accord
10. Les médecins possèdent des connaissances essentielles pour m’aider à guérir que les physiothérapeutes en pratique avancée n’ont pas.
    1. Tout à fait en accord
    2. En accord
    3. Ni accord ni en désaccord
    4. Pas d’accord
    5. Pas du tout d’accord

48

1. Auriez-vous confiance au physiothérapeute en pratique avancée pour qu’il prenne des décisions adéquates pour votre santé?
   1. Pas confiant du tout
   2. Pas très confiant
   3. Confiance modérée
   4. Très confiant
   5. Extrêmement confiant
2. Les traitements donnés par un physiothérapeute en pratique avancée seraient :
   1. Très sécuritaires
   2. Sécuritaires
   3. Peu sécuritaires
   4. Pas du tout sécuritaires

30. Le  physiothérapeute en pratique avancé me réfèrerait à un médecin si ma condition de santé l’exigeait.

1. Pas confiant du tout
2. Pas très confiant
3. Confiance modérée
4. Très confiant
5. Extrêmement confiant

31. De façon globale, la pratique avancée en physiothérapie est un phénomène :

1. Très favorable
2. Favorable
3. Ni favorable ni défavorable
4. Peu favorable
5. Très peu favorable

**Partie 3 : Questions démographiques**

La dernière section comporte des questions d’ordre général qui nous permettront de comparer vos réponses à ceux d’autres personnes ayant des caractéristiques semblables aux vôtres.

32. Quel est votre sexe?

A. Homme B. Femme

33. Dans quelle catégorie d’âge êtes-vous?

1. 18-24 ans
2. 25-29 ans
3. 30-34 ans
4. 35-39 ans
5. 40-44 ans
6. 45-49 ans
7. 50+ ans

34. Quelle est votre occupation?

1. Étudiant
2. Professeur
3. Chercheur
4. Soutien technique
5. Membre de la direction
6. Autre : Précisez :

35. Quel est votre statut matrimonial?

A. Célibataire B. Marié C. Divorcé D. Veuf

36. Quel est votre niveau d’étude complété?

1. Primaire
2. Secondaire
3. Cégep
4. Baccalauréat
5. Cycle supérieur

37. Quelle est votre langue maternelle ?

A. Français

B. Anglais

C. Autre
