# Supplementary material for: Acceptability of physiotherapists as primary care practitioners and advanced practice physiotherapists for care of patients with musculoskeletal disorders: a survey of a university community within the province of Quebec
Source: BMC Musculoskelet Disord. 2016 Sep 21;17:400. doi: 10.1186/s12891-016-1256-8 (PMC5031271; doi:10.1186/s12891-016-1256-8)
Supplement: Additional file 2: — English translation of the survey. (DOCX 111 kb) [file 12891_2016_1256_MOESM2_ESM.docx]

**Appendix 2**

Context

This study is a quantitative survey study on the public’s perception of physiotherapy. All responses are confidential and no information that could allow your identification will be collected. The survey takes about 15 minutes to complete. The questionnaire will be divided into two sections. The first section will assess your perception and your level of knowledge of the physiotherapy profession. The emergence of physiotherapy in recent decades makes it now an important profession, and it is important to survey the population about their perception of the profession. The second part of the survey will assess your perception in relation to advanced practice physiotherapy in Québec. We see the emergence of new roles for physiotherapists in order to improve the accessibility and efficiency of health care for patients with osteoarthritis, tendinitis or back pain. The survey is conducted by a researcher from Laval University, Jean-Sébastien Roy in collaboration with master in physiotherapy students and a researcher from the University of Montreal, François Desmeules.

1. Do you work in the rehabilitation department or do you study in the physiotherapy program at Laval University?

A. Yes * Thank you for your response, but you are not eligible to participate in the study

B. No

***Part 1: Public Perception of Physiotherapists as Primary Care Providers for the Treatment of Various Musculoskeletal Problems.***

2. Have you ever used the services of a physiotherapist or a physical rehabilitation therapist?

1. Yes
2. No * Go to 7
3. I do not know * Go to 7

3. You have received these physiotherapy treatments (check all that apply):

1. In a private setting
2. In a hospital
3. Via a CLSC (Québec’s community health center)
4. In a rehabilitation center
5. During a sporting activity
6. Other setting: Please specify: ___________

4. When was your last visit in physiotherapy?

1. During the course of the past year
2. Between one to two years ago
3. Between 2-5 years ago
4. Between 5-10 years ago
5. More than 10 years ago

5. What was your level of satisfaction following the treatments received in physiotherapy?

If you have received treatments for various health problems, consider your last course of treatment for a given health problem.

1. Very satisfied
2. Satisfied
3. Dissatisfied
4. Very dissatisfied
5. I do not know

6. There are two classes of professionals who can provide physiotherapy care and treatment: the physiotherapist who has a university level training or a physical rehabilitation therapist that has a college level training. Which of these professionals provided the treatment your received during your most recent visits?

1. Physical therapist
2. Physical rehabilitation therapist
3. Both
4. I do not know which professional was providing care and treatment

7. If you have ever needed to consult a physiotherapist or if you would need to do so in the future, how have you or would you proceed to select one ?

1. Upon the advice of your doctor
2. Upon the advice of a family member or friend
3. Based on proximity criteria (close to your home or your work)
4. Based on informations, advertising on the internet in the media or in the *Yellow Pages*
5. Other reasons. Please specify: ____________
6. I do not know

8. What is your opinion about the skills and competence of physiotherapists?

1. Very competent and skilled
2. Competent and skilled
3. Somewhat competent and skilled
4. Barely competent and skilled
5. I do not know

9. What is your opinion about the competence of physical rehabilitation therapists?

1. Very competent and skilled
2. Competent and skilled
3. Somewhat competent and skilled
4. Barely competent  and skilled
5. I do not know

10. Do you have confidence in the quality of the treatment you received, or you would received in physiotherapy?

1. Very confident
2. Confident
3. Somewhat confident
4. Very little confidence
5. I do not know

11. In your opinion, what are the health problems that a physiotherapist can manage (assess and treat)? (Check all the problems that apply.)

1. Musculoskeletal problems (Eg sprain, tendinitis, back and neck pain)
2. Urinary incontinence (in elderly people or post pregnancy)
3. Balance disorders (frequent falling secondary to a neurological condition)
4. Neurological disorders (stroke)
5. Walking disorders
6. Respiratory diseases (pneumonia)
7. Pediatric problems (developmental disorders in children)

12. If you have back or neck problems, a sprain, a tendinitis or if you have muscle or joint pain, do you believe that a **physiotherapist can make a** diagnosis equivalent to the one of a family doctor or an emergency doctor?

1. Yes, physiotherapists can make diagnoses as valid and precise as doctors do
2. No, I think that the diagnoses of doctors are more valid and precise
3. No, I think that the diagnoses of physiotherapists are more valid and precise
4. I do not know

13. If you have back or neck problems, a sprain, a tendinitis or if you have muscle or joint pain, do you think it is always necessary to have an X-ray, a magnetic resonance imaging test or another radiological test to make a valid diagnosis?

1. Yes
2. No, these tests are not always necessary
3. No, but I prefer to have a confirmation of the diagnosis with one of these tests.
4. I do not know

14. If you have back or neck problems, a sprain, a tendinitis or if you have muscle or joint pain, do you believe it is always necessary to take prescription drugs to effectively treat these problems?

1. Yes
2. No, prescription drugs are not always necessary
3. No, but it speeds up the healing process
4. I do not know

15.Before a physiotherapist evaluates your condition and treats you, a medical consultation is:

1. Absolutely essential
2. Essential
3. Somewhat essential
4. Not essential at all

16. According to you, is a medical referral mandatory to see a physiotherapist in a private clinic?

1. Yes
2. No
3. I do not know

17. Which health care provider do you or would you consult **first** for back or neck pain?

1. Physician
2. Chiropractor
3. Kinesiologist
4. Physiotherapist
5. Massage therapist
6. Osteopath
7. Other. Please specify: ________________

18. Which health care provider do you or would you consult **first** for a sprain of the arms or legs

1. Physician
2. Chiropractor
3. Kinesiologist
4. Physiotherapist
5. Massage therapist
6. Osteopath
7. Other. Please specify: ________________

19. Which health care provider do you or would you consult **first** for a tendinitis, muscle or joint pain in the arms or legs?

1. Physician
2. Chiropractor
3. Kinesiologist
4. Physiotherapist
5. Massage therapist
6. Osteopath
7. Other. Please specify: ________________

***Part 2: Public Perception of Advanced Practice Physiotherapists***

The second part of the questionnaire focuses on advanced practice in physiotherapy. This is a new model of care in which **physiotherapists receive additional training** and are allowed to perform acts normally reserved to physicians such as the right to make a medical diagnosis, patient assessment to determine whether a surgery is necessary, ordering of imaging tests (e.g.: radiography and MRI) and in some cases prescribe / inject medication in patients that have back, neck, muscle or joint pain or have a sprain or tendinitis. Providing more autonomy to the physiotherapists allows to reduce the burden on the health care system. These advanced practice physiotherapists, following additional training, could be implented in Québec in the near future. This is a phenomenon similar to that of nurse practitioners, often called super-nurses.

The following questions are related to advanced practice physiotherapy for the management and treatment of patients that have back, neck, muscle or joint pain or have a sprain, a tendinitis, osteoarthritis or other musculoskeletal problems (i.e. of muscles, bones, joints or tendons)

The following questions ask you to indicate your level of confidence in respect to various aspects of advanced practice physiotherapists, if they were implemented here in the province of Québec.

20. The advanced practice physiotherapist would determine your diagnosis and in most cases you would not meet a physician. What would be your level of confidence in an advanced practice physiotherapist in this role?

1. Not at all confident
2. A little confident
3. Moderately confident
4. Very confident
5. Extremely confident
6. I do not know

21. The advanced practice physiotherapist would prescribe radiological tests deemed necessary to evaluate your condition (X-ray, magnetic resonance or other radiological tests). What would be your level of confidence in an advanced practice physiotherapist in this role?

1. Not at all confident
2. A little confident
3. Moderately confident
4. Very confident
5. Extremely confident
6. I do not know

22. The advanced practice physiotherapist would determine if you need surgery for treating your musculoskeletal problems (muscles, bones, joints or tendons) and then refer you to meet with the surgeon who would determine the required surgery. What would be your level of confidence in an advanced practice physiotherapist in this role?

1. Not at all confident
2. A little confident
3. Moderately confident
4. Very confident
5. Extremely confident
6. I do not know

23. The advanced practice physiotherapist could prescribe certain prescription drugs like anti-inflammatory medication. What would be your level of confidence in an advanced practice physiotherapist in this role?

1. Not at all confident
2. A little confident
3. Moderately confident
4. Very confident
5. Extremely confident
6. I do not know

24. The advanced practice physiotherapist could perform injections in muscles or joints. What would be your level of confidence in an advanced practice physiotherapist in this role?

1. Not at all confident
2. A little confident
3. Moderately confident
4. Very confident
5. Extremely confident
6. I do not know

25. As the physiotherapist would be the only primary health care provider seen for evaluation and treatment and I would not meet a physician, this would allow me to spend less time waiting for an appointment or for treatment.

1. Strongly agree
2. Agree
3. Neither agree nor disagree
4. Disagree
5. Strongly disagree

26. As the physiotherapist would be the only primary health care provider seen for evaluation and treatment and I would not meet a physician, this would allow a shorter hospital length of stay.

1. Strongly agree
2. Agree
3. Neither agree nor disagree
4. Disagree
5. Strongly disagree

27. Physicians have essential knowledge to help me heal that advanced practice physiotherapists therapists do not have.

1. Strongly agree
2. Agree
3. Neither agree nor disagree
4. Disagree
5. Strongly disagree

28. How confident are you that the advanced practice physiotherapists would take adequate decisions for your health?

1. Not at all confident
2. A little confident
3. Moderately confident
4. Very confident
5. Extremely confident
6. I do not know

29. Treatments given by an advanced practice physiotherapists would be:

1. Very safe
2. Safe
3. Somewhat safe
4. Not safe at all

30. The advanced practice physiotherapists would refer me to a physician if required by my health condition

1. Not at all confident
2. A little confident
3. Moderately confident
4. Very confident
5. Extremely confident
6. I do not know

31. Overall, the advanced practice physiotherapy is a phenomenon:

1. Very favorable
2. Favorable
3. Neither favorable nor unfavorable
4. Unfavorable
5. Very unfavorable

**Part 3: Demographic Questions**

The last section includes general questions that will allow us to compare your answers with those of other people with characteristics similar to yours.

32. What is your sex?

A. Male

B. Female

33. What age group do you belong to ?

1. 18-24 years
2. 25-29 years
3. 30-34 years
4. 35-39 years
5. 40-44 years
6. 45-49
7. 50+ years

34. What is your occupation?

1. Student
2. Professor
3. Researcher
4. Support or technical personnel
5. Managing position
6. Other: Please describe:___________

35. What is your marital status?

A. Single

B. Married

C. Divorced or separated

D.Widowed

36. What is your highest level of education completed?

1. Primary school
2. Secondary school
3. College
4. Undergraduate
5. Postgraduate

37. What is your native language?

A. French

B. English

C. Other
